# Measurement of Ghrelin as a Marker of Appetite Dysregulation in Cats with and without Chronic Kidney Disease

**DOI:** 10.3390/vetsci10070464

**Published:** 2023-07-14

**Authors:** Katelyn Brusach, Sarah Lorbach, Jessica Quimby, Eline Nijveldt, Rene Paschall, Hannah Kinsella, Valerie Parker, Ramiro Toribio

**Affiliations:** Department of Veterinary Clinical Sciences, College of Veterinary Medicine, The Ohio State University, Columbus, OH 43210, USA; brusach.1@osu.edu (K.B.); lorbach.10@osu.edu (S.L.); nijveldt.1@osu.edu (E.N.); paschall.7@osu.edu (R.P.); kinsella.25@osu.edu (H.K.); parker.888@osu.edu (V.P.); toribio.1@osu.edu (R.T.)

**Keywords:** feline, chronic renal disease, acylated ghrelin, desacyl ghrelin, appetite, acidification, aprotinin

## Abstract

**Simple Summary:**

Cats with chronic kidney disease (CKD) frequently suffer from weight loss and inadequate caloric intake due to poor appetite. Ghrelin is a key hormone involved in the regulation of appetite, and it circulates in two forms: acylated and desacyl ghrelin. Acylated ghrelin is associated with initiating and stimulating appetite, whereas desacyl ghrelin is considered anorexigenic. To investigate appetite regulation in cats, this study compared plasma acylated, total, and calculated desacyl ghrelin concentrations in cats with and without CKD. The results demonstrate that cats with CKD have increased desacyl and total ghrelin concentrations in comparison to normal cats, consistent with dysregulation of appetite. This increase was correlated with disease severity.

**Abstract:**

Appetite abnormalities and weight loss are important comorbidities in the treatment of chronic kidney disease (CKD) in cats. Ghrelin, a key hormone involved in the regulation of appetite and metabolism, is a potential marker of appetite dysregulation in cats with CKD. The aim of this study was to compare the plasma concentrations of acylated, desacyl, and total ghrelin in normal cats and cats with CKD. Storage methodology was investigated prior to evaluating ghrelin concentrations in normal and CKD cats to facilitate clinical sample collection. Twelve normal cats and twelve cats with CKD were enrolled. Plasma acylated and total ghrelin concentrations were measured using radioimmunoassay. Desacyl ghrelin was calculated (total ghrelin minus acylated ghrelin). Cats with CKD had significantly increased total ghrelin and calculated desacyl ghrelin concentrations in comparison to normal cats (*p* < 0.0001 and *p* = 0.0001). There was no significant difference in active ghrelin concentrations between groups. Both total ghrelin and calculated desacyl ghrelin were significantly correlated with serum creatinine concentrations (*p* < 0.0001, r = 0.70 and *p* < 0.0001, r = 0.73). Elevated plasma desacyl ghrelin concentrations in cats with CKD provides evidence for dysregulation of appetite in feline CKD.

## 1. Introduction

Abnormal appetite and weight loss are significant comorbidities observed in cats with chronic kidney disease (CKD), with approximately 21–92% of caregivers reporting changes in their CKD cats’ appetite [1,2,3]. Low body weight is also common, affecting 36–81% of feline CKD patients, and is associated with a poorer prognosis [3]. Additionally, appetite is perceived as a component of quality of life in cats, and in many cases, a poor appetite is a factor in the decision to euthanize [4]. Although the cause of weight loss may be multifactorial, impaired nutritional intake is undoubtedly a major component [5]. An investigation into the hormones influencing appetite in cats with CKD is necessary to better understand the pathophysiology of appetite regulation. Management of appetite represents an important therapeutic target, offering the possibility to improve survival and quality of life in these patients.

Appetite regulation involves a balance of orexigenic (appetite-stimulating) and anorexigenic (satiety-inducing) substances. Ghrelin is a key hormone involved in the regulation of appetite and metabolism [6,7]. In healthy humans, approximately 10% of ghrelin circulates as acylated ghrelin (AG), while the majority circulates as desacyl ghrelin (DG) [8,9,10]. AG is associated with initiating and stimulating appetite, whereas DG is considered anorexigenic [6,7]. Ghrelin is eliminated via the kidneys, and in patients with impaired kidney function, AG concentrations have been shown to remain unchanged while total ghrelin (TG) accumulates [11]. In pediatric human patients with kidney disease, DG is positively correlated with serum creatinine concentrations [12]. Therefore, measurement of ghrelin might provide a deeper understanding of inappetence in CKD, as well as provide support for its potential as a therapeutic. 

Acylation with octanoic acid on the third serine amino acid is necessary for binding of ghrelin to the growth hormone secretagogue receptor 1a and its biological activity [8]. However, the octanoyl group can be disrupted during sample collection, processing, or storage, resulting in degradation of AG to DG. This has the potential to confound the comparison of circulating AG and DG concentrations, for example in kidney disease [10]. In humans, AG is better preserved throughout sample collection and storage when EDTA is used as an anticoagulant with a protease inhibitor (aprotinin) [10]. Additionally, acidification of the sample results in improved stability of the acyl modification [10,13]. This methodology has not been fully investigated or universally employed in cats. Improving the stability of ghrelin forms in storage would facilitate the feasibility of clinical sample collection. 

The primary objective of this study was to compare the concentrations of acylated, desacyl, and total ghrelin in the plasma of normal cats and cats with CKD. To facilitate more accurate measurement of AG versus DG for this aim, a secondary objective was to compare the effect of sample processing and storage on the recovery of AG in the plasma of normal cats. Given the possible effect of kidney dysfunction on the accumulation of anorexigenic compounds, the characterization of ghrelin in cats with CKD will provide valuable information to better understand appetite dysregulation in disease. 

## 2. Materials and Methods

### 2.1. Sample Storage Optimization

#### 2.1.1. Cats

Five normal cats were enrolled. All cats were screened for general health. Cats were excluded if any clinical signs consistent with chronic illness were reported by the owner. No medications were allowable other than routine parasite control and veterinary visit-associated gabapentin. Complete blood counts, chemistry tests, urinalyses, blood pressure tests, and total thyroxine concentration analyses were performed to confirm health status (serum creatinine < 1.6 mg/dL and USG > 1.035). The study was approved by The Ohio State University Institutional Animal Care and Use Committee (IACUC #2020A00000058), and written informed consent was obtained from the owner or legal guardian of all cats described in this study for the procedures undertaken.

#### 2.1.2. Sample Collection

**Figure 1:**
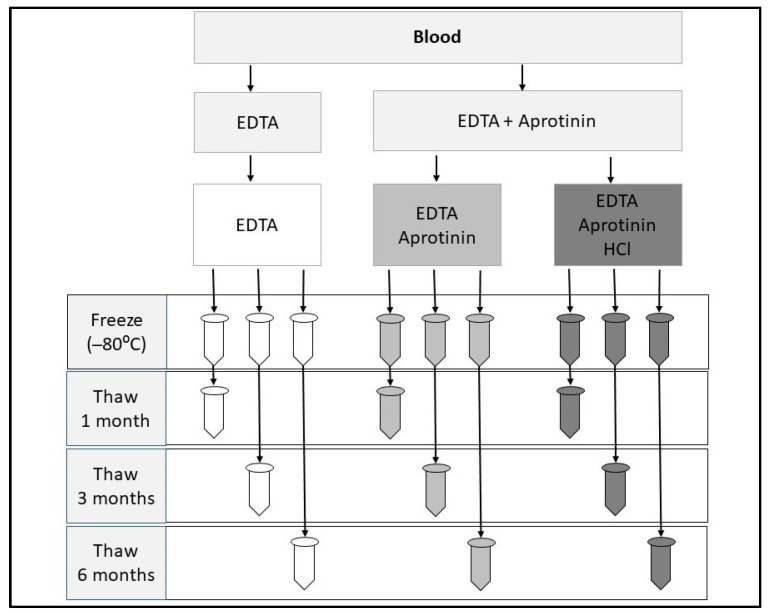
Schematic illustrating sample collection and processing.

Cats were presented after a 10 h fast. Blood was drawn (4.5 mL) and immediately distributed into pre-chilled blood tubes: 1.5 mL into a tube containing EDTA (2 mg/mL blood; Covidien) and 1.5 mL each into 2 tubes containing EDTA + Aprotinin (500 KIU/mL blood; Bovine Lung, EMD Millipore 616370). Tubes were kept chilled, and samples were immediately centrifuged at 1000× *g* at 4 °C for 10 min. Three aliquots of 240 µL of plasma were collected and processed in 3 ways: EDTA only, EDTA + Aprotinin (EA), and EDTA + Aprotinin acidified (EAH) (Figure 1). For acidification, the plasma from one EDTA + Aprotinin tube was taken, and 1N hydrochloric acid was titrated until a pH of 4.0 (3.9–4.1) was reached (average 17–24 µL). Samples were divided into 1-, 3-, and 6-month aliquots and stored at −80 °C until analysis. At the time of analysis, the 240 µL aliquot was split into two replicates (120 µL each) for the radioimmunoassay (RIA).

#### 2.1.3. Sample Measurement

Radioimmunoassay (Human ghrelin (active) RIA; Sigma-Aldrich; catalogue no. GHRA-88HK) was performed in duplicate at 1 month, 3 months, and 6 months after sample collection to measure plasma AG concentrations. This assay was performed according to manufacturer guidelines and has been previously validated for use in cats [14]. The structure of feline ghrelin is identical to rat and human ghrelin except for one amino acid [15]. Briefly, assay buffer was added to all tubes excluding blanks. Standards and quality controls were added to their designated tubes and diluted accordingly. Samples were then added prior to the addition of ghrelin antibody and incubated at 4 °C for 20–24 h. After incubation, iodine-125 ghrelin tracer was added and incubated at 4 °C for 22–24 h. The following day, precipitating reagent was added, followed by a 20 min incubation at 4 °C for 20 min. Manufacturer guidelines state to drain tubes between 15 and 60 s (consistent between racks) and to blot excess supernatant from the lip of tubes leaving the count pellet. While performing the third assay (Month 6), a minor change in methodology was performed. A timer was used to standardize the decanting of supernatant from the centrifuged tubes and a simpler approach was taken to blot excess liquid from the lip of the tubes. Samples were read using a gamma counter (Wallac Wizard 1470 Gamma Counter, Perkin Elmer, Shelton, CT, USA). Intra-assay coefficient of variation (using replicates from all sample processing types) was 10.4%, 5.3%, and 5.2% at 1 month, 3 months, and 6 months, respectively. Inter-assay coefficient of variation (average of all 3 time points) was 21.2%, 22.7%, and 25.9% for EDTA, EA, and EAH samples, respectively.

#### 2.1.4. Statistical Analysis

Statistical analysis was performed using Prism (GraphPad Software 9.1.1, San Diego, CA, USA). Data were not normally distributed, and Friedman’s test was used to compare AG concentrations between sample storage methods with Dunn’s post hoc comparison. For statistical analysis, plasma concentrations of AG in EDTA and EA were proportionally normalized to that of EAH. Data are presented as median and range. Statistical comparison between time points was not performed due to the minor change in methodology at the 6-month time point that could have affected results.

### 2.2. Ghrelin in Cats with and without CKD

#### 2.2.1. Cats

Cats with stable International Renal Interest Society (IRIS) Stage 2–3 CKD (serum creatinine ≥ 1.6 mg/dL, USG < 1.035) (*n* = 12) and normal control cats (serum creatinine < 1.6 mg/dL, USG > 1.035) (*n* = 12) were enrolled. Complete blood count, biochemistry profile, total thyroxine concentration, urinalysis, and blood pressure were used to determine enrollment eligibility. Patients with chronic gastrointestinal signs, diabetes mellitus, hyperthyroidism, other uncontrolled systemic illness, or complications of CKD (urinary tract infection/pyelonephritis, hypertension, etc.) were excluded. Obese (body condition score (BCS) 8/9 or 9/9) or underweight (BCS ≤ 4/9) cats were excluded from enrollment. No medications were allowable for normal cats other than routine parasite control. CKD cats could not have received an appetite stimulant in the last week prior to participation in the study. It was not anticipated that age-matching would be possible, but effort was made to enroll older normal control cats. The study was approved by The Ohio State University Institutional Animal Care and Use Committee (IACUC #2021A00000065) and written informed consent was obtained from the owner or legal guardian of all cats described in this study for the procedures undertaken.

#### 2.2.2. Appetite Questionnaire

Caregivers of both normal cats and cats with CKD filled out a simple questionnaire related to appetite and food behaviors at the time of sample collection (Box 1).

Box 1 **Appetite Questionnaire.**

**1. Is your cat’s appetite:** Increased   Decreased   Unchanged

**2. How many days a week do you feel your cat experiences a decrease appetite?**

0      1       2       3       4       5       6       7

**3. How many days a week do you feel you have to encourage your cat to eat?**

0      1       2       3       4       5       6       7

**4. Which of the following has your cat exhibited in the past week:**

Eating less

Not as interested in food

Not eating as long

Not eating as quickly

Less excited to be fed

Not begging for food as much

Acts hungry but then turns away

Not enjoying food as much

None of these apply

**5. Please describe your cat’s appetite in the past few days using this scale (0–4)**

0: 0–25% of food consumed

1: 25–50% of food consumed

2: 50–75% of food consumed

3: 75–100% of food consumed

4: 100% of food consumed

**7. Have you felt the need to rotate or offer a variety of foods to encourage your cat to eat?** Yes No

#### 2.2.3. Sample Collection and Preparation

Cats were fasted for at least 10 h prior to blood collection. Immediately following venipuncture, 500 µL of blood was deposited into a chilled EDTA (2 mg/mL blood; Covidien) tube containing 10.2 µL of aprotinin (500 KIU/mL blood; Bovine Lung, EMD Millipore 616370). Samples were chilled while in transport to the laboratory for further processing. Blood was immediately centrifuged at 4 °C at 1000× *g* for 10 min. Plasma (approximately 250 µL) was immediately transferred to a cryovial using a pipette and then acidified to a pH of 4 (range 3.9–4.1) with 1N HCl. Given normal variation in the individual plasma samples in both volume and pH, the volume of HCl was not standardized but gradually added in 1–10 uL increments until target pH was achieved. Upon completion of acidification, cryovials were stored at −80 for no more than three months until analysis.

#### 2.2.4. Sample Analysis

Radioimmunoassays (Human ghrelin (total) RIA; GHRT-89HK, and Human ghrelin (active) RIA; GHRA-88HK, Sigma-Aldrich) were used to measure AG and TG. Subsequently, DG was calculated from these values. All CKD and normal cat samples were analyzed in duplicate in the same assay run. Protocols were followed according to assay instructions (described above). Tubes were decanted using a timer to standardize the duration they were inverted and, then, were blotted to remove excess liquid from the lip of the tubes. Intra-assay coefficient of variation (using replicates from all samples type) was 4.1% for TG assay and 14.0% for AG assay.

#### 2.2.5. Statistical Analysis

An a priori power calculation was performed to determine how many cats with and without CKD would be needed. Human studies indicate significant differences in TG concentrations between normal controls (983.1 +/− 580.5 pg/mL) and those with CKD (2387 +/− 1401 pg/mL) [11]. A power calculation for TG was performed in JMP Pro 14.0 using the means of the two groups and the more conservative SD (140 pg/mL) of the two groups (as opposed to averaging the two). With alpha 0.05, 12 cats per group would be required for 80% power.

Statistical analysis was performed using GraphPad Prism (GraphPad Software 9.1.1, San Diego, CA, USA). AG concentrations were subtracted from TG concentrations to calculate DG concentration (TG-AG=DG). Serum creatinine concentration, body weight, age, TG, AG, and DG concentrations were compared between cats with and without CKD using a Mann–Whitney test. Correlations between serum creatinine and TG, and AG and DG concentrations were assessed using Spearman rank. 

## 3. Results

### 3.1. Sample Storage Optimization

#### 3.1.1. Cats

Of the five normal cats enrolled, three were spayed female and two were neutered males, all mixed breeds. The median age was 2.3 years (range 1.8–9.3 years), and the median weight was 5.1 kg (range 3.6–6.6 kg). All cats had a body condition score between 5 and 7/9 with normal muscle condition.

#### 3.1.2. Radioimmunoassay Results

The median (range) plasma AG concentrations after storage for 1, 3, and 6 months are detailed in Table 1. One cat was determined to be an outlier based on a value lower than 3.5 standard deviations from the median and was not included in the 1-month storage data. All five cats were included in the 3-month and 6-month storage data. 

**Figure 2:**
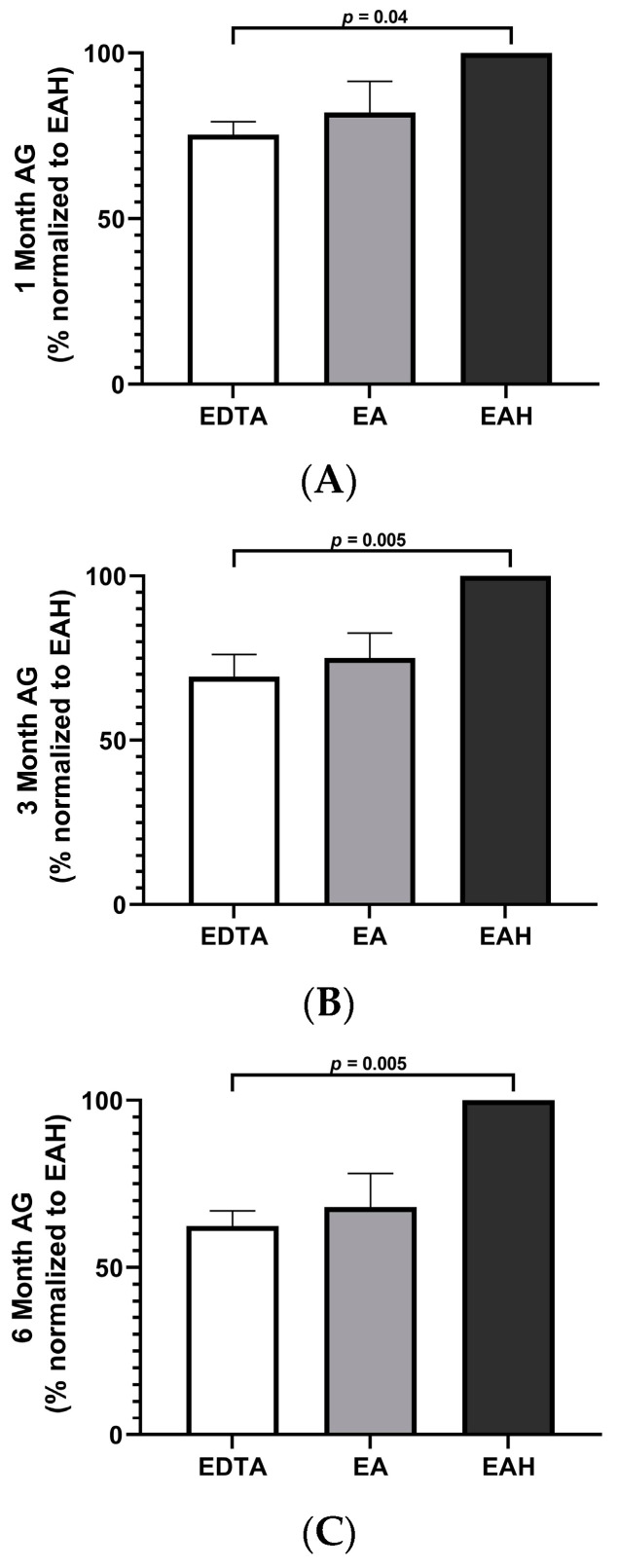
Plasma concentrations of acylated ghrelin in EDTA and EDTA with aprotinin (EA) in normal cats after (**A**) 1 month, (**B**) 3 months, and (**C**) 6 months of storage proportionally normalized to samples processed with acidification.

When the plasma AG concentrations in EDTA and EA were proportionally normalized to that of EAH, the EAH aliquots had significantly higher yield than the aliquots processed in EDTA alone at all three times points (Table 2 and Figure 2). There was no statistically significant difference in the yield of AG in EDTA versus EA, nor in EA versus EAH, despite subjective differences. 

### 3.2. Ghrelin in Cats with and without CKD

#### 3.2.1. Cats

Twelve normal cats and twelve cats with CKD were enrolled (Table 3). Of the normal cats, two were spayed females and ten were neutered males, all mixed breeds. The median age of the normal cats was 5.5 years, (range 3.3–14.3 years) and the median weight was 5.0 kg (range 3.3–6.3 kg). All normal cats had a BCS of 5–7/9 with normal muscle condition. The median serum creatinine concentration was 1.3 (range 0.7–1.6 mg/dL). One 4-year-old normal cat had a serum creatinine of 1.6 mg/dL (USG 1.040), which was 1.3 mg/dL on recheck so this cat remained in the normal group. 

Cats with CKD consisted of eight IRIS Stage 2 cats and four IRIS Stage 3 cats. The median serum creatinine concentration was 2.4 (range 1.6–3.4). Of the CKD cats, five were spayed females and seven were neutered males. All were mixed breeds, with the exception of one Siamese. The median age of the CKD cats was 12.3 years (range 10–19 years), and the median weight was 3.8 kg (range 2.5–5.5 kg). All CKD cats had a BCS of 4–7/9, and their muscle condition scores varied. Six cats had no muscle atrophy, three had mild muscle atrophy, two had moderate muscle atrophy, and one had severe muscle atrophy.

#### 3.2.2. Appetite Questionnaire

Of the 12 cats with CKD, 5/12 (42%) were reported to have decreased appetite, as observed by the caregiver. Caregivers felt that they needed to encourage their cat to eat two days a week (2/5 cats), four days a week (2/5 cats), and seven days a week (1/5 cats). Signs of decreased appetite reported in cats in the CKD group included: not eating as long (4/12), eating less (5/12), not as interested in food (2/12), acting hungry and then turning away (2/12), and not eating as quickly (1/12). Caregivers of 7/12 cats with CKD (58%) felt the need to rotate foods or offer a variety of foods to encourage their cat to eat. Four caregivers described their cat as consuming 100% of food offered, six caregivers described their cat as consuming 75–100% of food offered, one caregiver described their cat as consuming 50–75% of food offered, and one caregiver described their cat as consuming 0–25% of food offered. 

None of the caregivers of the twelve normal cats reported a decreased appetite. Only one caregiver felt they needed to encourage their normal cat to eat three to four days in the week. However, this caregiver attributed the cat’s decreased appetite to a new dog in the house. None of the normal cat caregivers felt the need to rotate or offer a variety of foods to encourage their cat to eat. All caregivers of normal cats described their cats as consuming 100% of food offered.

#### 3.2.3. Radioimmunoassay Results

**Figure 3:**
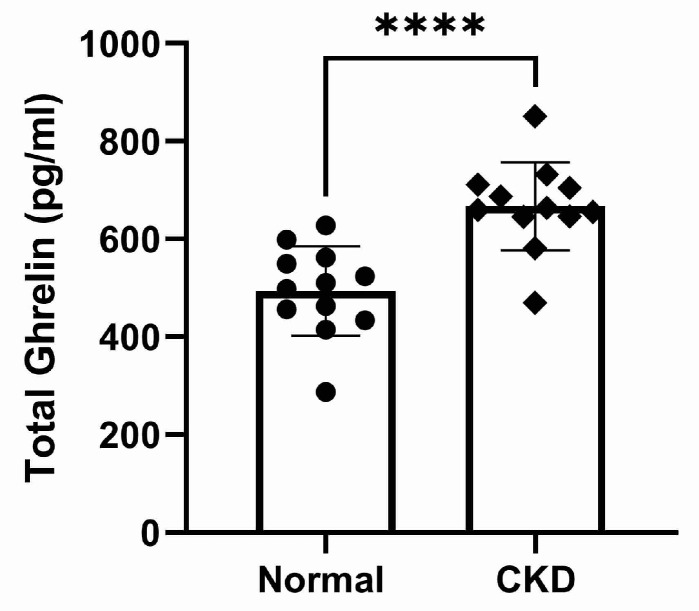
Total ghrelin plasma concentrations (pg/mL) in normal and CKD cats. Cats with CKD had significantly higher total ghrelin plasma concentrations compared to normal cats (*p* < 0.0001).

**Figure 4:**
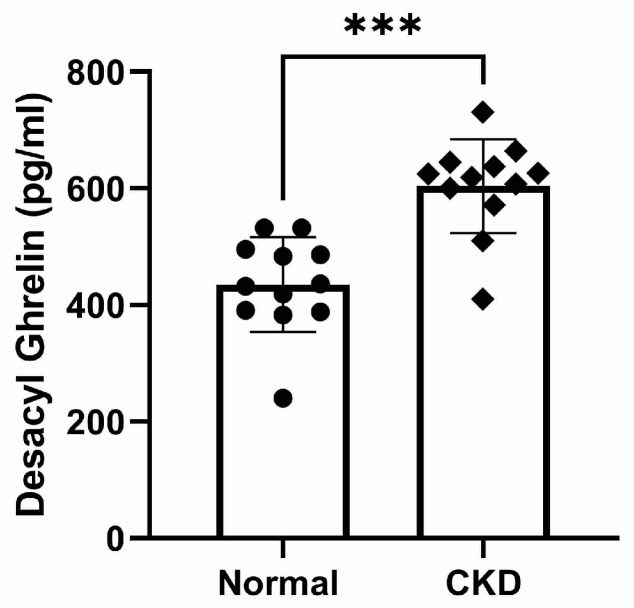
Calculated desacyl ghrelin plasma concentrations (pg/mL) in normal and CKD cats (*p* < 0.0001). Cats with CKD had significantly higher desacyl ghrelin plasma concentrations compared to normal cats.

**Figure 5:**
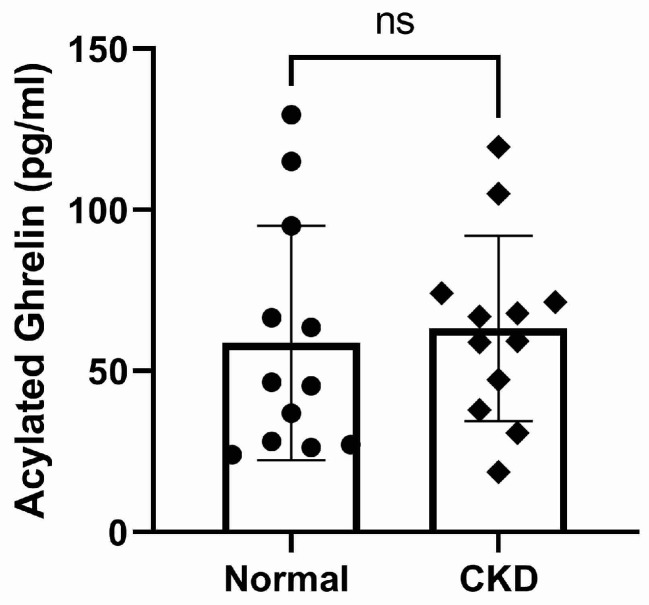
Active ghrelin plasma concentrations (pg/mL) in normal and CKD cats. There was no significant difference between active ghrelin plasma concentrations in CKD cats compared to normal cats (*p* < 0.41).

**Figure 6:**
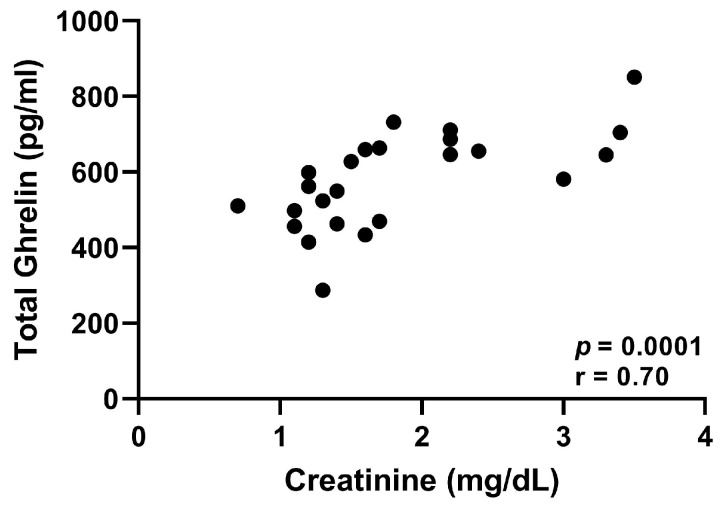
Serum creatinine (mg/dL) vs. plasma total ghrelin concentration (pg/mL) of all 24 cats. Serum creatinine significantly correlated with plasma total ghrelin (*p* < 0.0001, r = 0.70).

**Figure 7:**
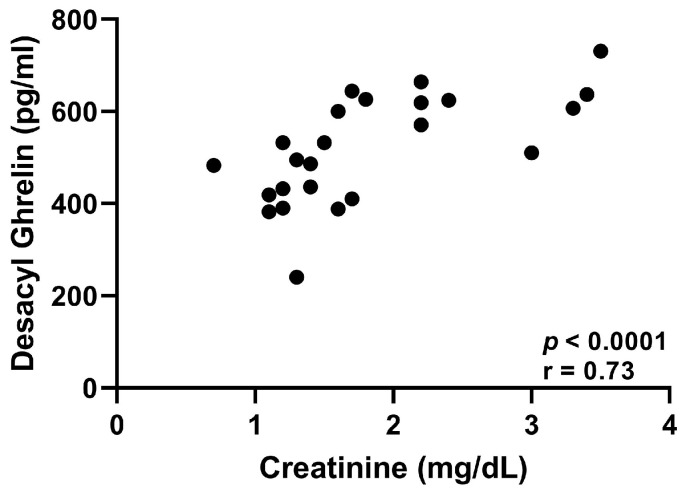
Serum creatinine (mg/dL) vs. plasma desacyl ghrelin concentrations (pg/mL) of all 24 cats. Serum creatinine significantly correlated with plasma desacyl ghrelin (*p* < 0.0001, r = 0.73).

The plasma concentrations of TG and calculated DG were significantly higher in cats with CKD compared to normal cats (*p* < 0.0001, Figure 3 and *p* = 0.0001, Figure 4, respectively). There was no significant difference in AG concentration between normal and CKD cats (Figure 5). There was a significant positive correlation between both TG and calculated DG and serum creatinine concentrations (*p* = 0.0001, r = 0.70, Figure 6, and *p* < 0.0001, r = 0.073, Figure 7, respectively).

## 4. Discussion

As hypothesized, DG and TG plasma concentrations were significantly increased in cats with CKD compared to normal cats. These findings support that there is hormonal dysregulation of appetite in cats with CKD. Furthermore, the positive correlation observed between both TG and calculated DG and serum creatinine concentrations supports the correlation between decreased kidney function and accumulation of anorexigenic compounds. This may provide some explanation as to why an increase in incidence of inappetence is observed in cats with CKD. AG did not differ between groups, suggesting that no compensatory increase in orexigenic signal occurs in CKD despite an increase in anorexigenic compounds.

These findings are similar to those in other species. In a study of human dialysis patients, plasma DG concentrations were found to be significantly higher than those in non-dialysis patients, yet no significant difference in AG was detected between groups [16]. A study of human pediatric patients found that TG concentrations were higher in CKD and dialysis patients compared to both controls and kidney transplant recipients [11]. Although plasma concentrations of AG were not significantly different between groups in this study, the ratio of AG to TG was significantly lower in CKD and dialysis patients, and this discrepancy was considered to be due to an increase in DG [11]. In another study in human pediatric patients with CKD, DG was significantly higher in CKD patients than that in controls and was negatively correlated with weight-standard deviation score (SDS), BMI-SDS, and percent fat mass [12]. Additionally, DG was positively correlated with creatinine and BUN, and inversely correlated with eGFR, and there was a trend towards improvement after kidney transplantation [12]. Several additional studies have reported on the relationship between declining kidney function and increasing DG concentrations [16,17,18,19,20,21]. This relationship has not been observed for AG, and thus, it has been presumed that it undergoes little renal clearance [22]. 

The relationship between increased DG concentrations and abnormalities in appetite is even more pertinent to appetite dysregulation in CKD. At least two studies have documented that DG concentrations are significantly higher in anorectic hemodialysis patients in comparison to those in non-anorectic patients [23,24]. In our study, a majority of the cats with CKD had abnormalities in appetite whereas the normal cats did not. However, the effects of an anorexigenic compound such as DG versus other abnormalities associated with CKD is hard to isolate. Unfortunately, our study was inadequately powered to compare DG concentrations between cats with normal and decreased appetite within the CKD group. 

Manipulation of orexigenic and anorexigenic compounds may represent a viable therapeutic option in treating inappetence in cats with CKD [25]. Mice administered DG had significantly decreased food intake as compared to both the control group and those administered AG, who experienced a pronounced increase in consumption of food [6]. In a study performed in human patients, AG and a placebo were administered to twelve malnourished dialysis patients in a weeklong double-blinded randomized crossover study [26]. The results showed an immediate and significantly increased appetite and sustained a positive change in energy balance with daily treatment of AG [26]. Another randomized, double-blinded, crossover study administered subcutaneous ghrelin to nine human dialysis patients with malnutrition who showed significantly increased absolute energy intake compared with placebo [27]. While the administration of AG shows promising results in restoring hunger hormone regulation and increasing appetence, these treatments have not been thoroughly tested or investigated in cats. However, the ghrelin receptor agonist capromorelin has been used to manage unintended weight loss and inappetence in cats [28]. 

A limitation of measuring ghrelin in this CKD cat population was the distribution of IRIS stages. Only IRIS Stage 2 and Stage 3 cats could be identified for enrollment; therefore, Stage 1 and Stage 4 patients are not represented in these data. Although it would not be anticipated that Stage 1 cats would have significant appetite hormone dysregulation due to their early disease state, this still merits investigation. IRIS Stage 4 cats are likely to be most affected by an accumulation of anorexigenic compounds and appetite dysregulation. However, the necessary frequent use of appetite stimulants in this patient group, combined with the frequency of comorbidities, impacted the ability to enroll them in this study, as the aim was to measure appetite hormone concentrations unaffected by these medications and other concurrent disease. Assessing TG, DG, and AG concentrations across all IRIS stages in a larger group of CKD cats may be beneficial to more completely describe the influence of disease severity on appetite regulation. 

The influence of age or sex on ghrelin concentrations in sterilized older cats is unknown and could not be assessed in the current study. The distribution of sexes was uneven in the normal cat group, and the CKD cats were significantly older than the normal cats. Age-matching could not be performed between normal cats and cats with CKD due to the challenge of finding elderly cats unaffected by disease. However, in one study in elderly humans, serum ghrelin concentrations were significantly lower in comparison to that of younger individuals [29]. Additionally, the frail elderly had significantly lower serum ghrelin concentrations in comparison to the non-frail. These results are in contrast to the findings in our study whereupon TG was significantly higher in the older population of cats with CKD, supporting the hypothesis that the effect is attributable to poor renal clearance. 

An additional limitation to the study was the lack of availability of a RIA assay specific to DG. The understanding of relative concentrations of orexigenic AG versus anorexigenic DG is key to understanding the influence of kidney disease on these compounds. We therefore calculated DG from TG and AG based on previous studies, and this calculation may be an approximate. However, this approach was substantiated by previous literature describing the relationship between TG, AG, and DG [11,16,24].

Prior to pursuing the comparison of plasma ghrelin concentrations in cats with and without CKD, it was important to first optimize sample handling and storage methodologies. The results of the sample storage portion of this study indicated that regardless of storage time, the recovery of AG was consistently higher when feline plasma was processed with EDTA and aprotinin and was acidified to a pH of 4.0 compared to the use of EDTA only. This finding is consistent with previous studies in other species, although most studies have not assessed storage over 6 months [10,13,30]. In this study, samples were frozen immediately after processing and thawed only once prior to performing the assay, and a significant difference was still observed in the recovery of AG. Although a subjective improvement in yield was observed with acidification, there was no significant difference in AG between EA and EAH samples. The study may have been underpowered to detect a significant difference between these groups. Increased yield of AG was demonstrated with acidification at all time points. This processing technique can improve the feasibility of obtaining clinical samples and batching them for analysis. However, it does require appropriate equipment to perform acidification safely. 

High inter-assay variability was observed between assays performed over 6 months. The effect of degradation of plasma AG versus analytical variability cannot be distinguished. In addition, due to the minor methodology change at 6 months, samples were not statistically compared over time. 

Aprotinin, a protease inhibitor, has been shown to improve yield of AG when compared to samples stored with EDTA alone [10,13]. This study showed no significant difference in plasma concentrations between samples stored with EDTA alone versus EDTA with aprotinin. However, with the exception of one individual aliquot, a subjectively higher yield of AG in EA was observed as compared to EDTA. Additional studies with larger patient populations may identify a significant difference between these two storage methods. The samples in our study were processed within 30 min of collection and kept chilled throughout the process. It is also possible that expedient processing and lack of additional freeze–thaw cycles may have allowed for retention of AG and minimized the difference between EDTA and EA containing aliquots. In contrast, a study in dogs demonstrated samples stored with aprotinin for 1 month or less did not consistently improve the yield of AG compared to EDTA alone [31]. However, no AG was detected in their samples with HCl. Methods for preventing degradation of AG vary between species [30,32,33]. It is possible that to prevent degradation of AG, ideal protease inhibition should be based on species-specific differences. At this time, these have not been fully evaluated in cats. 

There was subjectively higher yield in the concentration of AG at Month 6 as compared to Month 3, and some of the sample concentrations in Month 1. While performing the third RIA (Month 6), a timer was used to standardize the decanting of supernatant from the centrifuge tubes and a simpler approach was taken to blot excess liquid from the lip of the tubes. Whether this could explain this difference is unknown. Alternatively, the difference observed between these timeframes could be attributed to analytical variability in the assay itself. The range of AG concentrations of cats in this study (23.7–134.2 pg/mL), irrespective of storage method, was similar to that of a previous study (10–97 pg/mL) in which the same assay was used with a similar processing method (anticoagulant + aprotinin) [34]. 

A limitation of the storage optimization aim was the small sample size which may have impacted our ability to detect significant differences in AG concentrations between EDTA and EA samples. However, the study was designed based on previous literature that had similar numbers of animals [10,13,31,33]. This study also did not have a control sample without any constituents (EDTA, aprotinin) for comparison to all three proposed methodologies. Given the known volatile nature of ghrelin and the need to conserve the amount of blood drawn, this was considered unnecessary to address the aim of the study. Additionally, assays were not run immediately after sample collection and were first performed after 1 month of storage. This methodology was made based on a combination of practicality and a precedent in the literature. In several studies where storage time is documented, 1 month of storage has been considered appropriate [30,33]. Performance of RIA requires specialized facilities that are not universally available. Given the inter-assay variability, we recommend caution when comparing data between institutions, especially when different assays have been employed. Future studies should target additional investigations in the stability in AG storage over time, and a more accurate assessment of inter-assay variability and methods that result in the most accurate recovery of AG. 

## 5. Conclusions

This study showed that the anorexigenic substances DG and total ghrelin were increased in cats with CKD compared to normal cats. This increase was correlated with disease severity and may be a putative explanation for abnormalities in appetite in this patient population. Processing samples using EDTA and aprotinin with acidification resulted in the highest yield of AG after storage and facilitated clinical collection of samples for batched analysis. 

## Figures and Tables

**Table 1:** Plasma concentrations of acylated ghrelin in normal cats (pg/mL) based on storage method over time. Data are displayed as median (range).

Storage Time	EDTA Only	EA	EAH
1 Month (*n* = 4)	65.6 * (51.6–90.4)	72.3 (52.9–100.2)	87.7 * (69.5–114.6)
3 Months (*n* = 5)	44.4 * (23.7–56.5)	44.8 (27.2–63.2)	61.3 * (36.1–85.3)
6 Months (*n* = 5)	60.1 * (37.0–83.3)	65.4 (44.8– 86.1)	94.5 * (52.4–136.6)

EA: EDTA plus aprotinin; EAH: EDTA plus aprotinin and acidification. * Significantly different at specified storage time comparing EAH to EDTA only (*p* < 0.05).

**Table 2:** Plasma concentrations of acylated ghrelin in normal cats (pg/mL) based on storage method over time with percentage normalized to samples processed with acidification. Data are displayed as median (range).

Storage Time	EDTA Only (%)	EA (%)	EAH (%)
1 Month (*n* = 4)	76.2 * (70.4–78.9)	86.4 (68.0–87.5)	100 *
3 Months (*n* = 5)	66.3 * (62.7–80.3)	75.3 (63.0–82.3)	100 *
6 Months (*n* = 5)	65.7 * (61.0–70.5)	74.2 (63.0–85.4)	100 *

EA: EDTA plus aprotinin; EAH: EDTA plus aprotinin and acidification. * Significantly different at specified storage time comparing EAH to EDTA (*p* < 0.05).

**Table 3:** Summary of normal and CKD cats enrolled, including age; weight; serum creatinine; and total, acylated, and calculated desacyl ghrelin plasma concentrations. Data are displayed as median (range).

	Normal (*n* = 12)	CKD (*n* = 12)
Age (years) *	5.5 (3.3–14.3)	12.3 (10.0–19.0)
Weight (kilograms) *	5.0 (3.3–6.3)	3.8 (2.5–5.5)
Serum Creatinine (mg/dL) *	1.3 (0.7–1.6)	2.4 (1.6–3.4)
Total Ghrelin (pg/mL) *	493.76 (414.68–627.66)	667.26 (469.23–851.0)
Acylated Ghrelin (pg/mL)	58.72 (24.02–129.62)	62.21 (18.7–119.68)
Desacyl Ghrelin (pg/mL) *	435.11 (410.29–731.31)	604.05 (240.40–532.57)

* Significantly different between groups (*p* < 0.05).

## Data Availability

The data presented in this study are available from the corresponding author upon request.
